# Superficial femoral vein transposition as a solution for hemodialysis vascular access

**DOI:** 10.1590/1677-5449.202101351

**Published:** 2022-10-03

**Authors:** Matheus Mannarino Carmo Silva Ribeiro, Eduardo Rodrigues, Alex Bezerra, Eric Paiva Vilela, Felipe Borges Fagundes, Cristiane Ferreira de Araújo Gomes, Cristina Ribeiro Riguetti Pinto, Carlos Eduardo Virgini-Magalhães

**Affiliations:** 1 Universidade do Estado do Rio de Janeiro – UERJ, Hospital Universitário Pedro Ernesto – HUPE, Rio de Janeiro, RJ, Brasil.

**Keywords:** arteriovenous fistula, chronic renal insufficiency, renal dialysis

## Abstract

The significant growth in the number of individuals dependent on hemodialysis for renal replacement therapy and unrestricted use of short and long-term catheters have challenged vascular surgeons in search of solutions for patients whose options for access via the upper limbs have been exhausted and for the increasing rates of central venous stenosis in these patients. When access via the upper limbs is impossible, exceptional techniques can be used and the lower limbs offer feasible alternative vascular access sites for hemodialysis. This article reports a case of superficial femoral vein transposition to make a loop arteriovenous fistula in a patient with no possibility of access via the upper limbs and presents a literature review on this technique that remains little used.

## INTRODUCTION

The last decade has seen significant growth in the number of people dependant on hemodialysis for renal replacement therapy (RRT). In Brazil, the number of chronic renal patients is rapidly approaching 150,000.^1^

There is no doubt that an autologous upper-limb arteriovenous fistula (AVF) is the first-choice access for hemodialysis. However, when access cannot be obtained via an upper limb, creation of a vascular access in a lower limb is a feasible option that can be a salvation.^2^

This project was approved by the Research Ethics Committee at the Institution, under decision number 4.783.675 (CAAE: 47569921.3.0000.5259). The objective of this article is to describe surgical creation of a vascular access for hemodialysis by superficial femoral vein transposition (SFVt) and present a review of the literature discussing the advantages and disadvantages of this technique that is still rarely used in our country.

### Part 1 – clinical situation

A 54-year-old male patient who had been on an RRT program for 21 years was referred for creation of a hemodialysis vascular access. He had a history of multiple upper limb accesses. He had undergone ligature of a basilic loop in July of 2020 because of central vein stenosis and ulceration with intermittent bleeding in the region of the puncture site, with no possibility of endovascular treatment. He stated that he had never had a provisional vascular access via a femoral vein and had been receiving dialysis via a long-term catheter implanted in the right subclavian vein for 2 months.

Vascular physical examination revealed multiple scars from previous AVFs in the upper limbs and no options for creation of a new access because of a lack of viable venous segments that could be used for the surgery and central vein stenosis on the left.

Faced with the impossibility of using the upper limbs, the dilemma would have to be solved by creating a vascular access via a lower limb, employing either autologous or prosthetic material.

### Part 2 – What was done

We decided to transpose the femoral vein, considering the better patency and lower risk of infection, when compared with a saphenous vein loop or a polytetrafluoroethylene (PTFE) graft, and the adequacy of the patient’s arterial and venous anatomy in the lower limbs, with strong pulses, normal ankle-brachial indices, and both deep and superficial vein systems free from abnormalities on Doppler ultrasonography.

**Figure 1:**
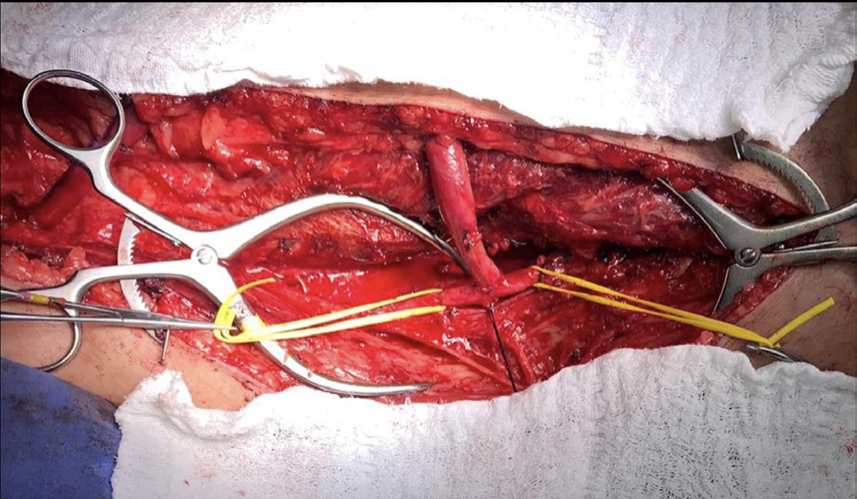
Surgical procedure. Detail showing the anastomosis between the artery and the superficial femoral vein.

The patient underwent superficial femoral vein transposition of the left lower limb in November of 2020, under spinal anesthesia, employing the technique described by Gradman et al.^2^ The great saphenous vein was identified with Doppler ultrasonography immediately before surgery, to avoid damage during access. The procedure was performed with no intraoperative complications, with a duration of around 2.5 hours (Figure 1).

On the first postoperative day, the popliteal and distal pulses were significantly diminished on palpation during physical examination, although the limb was well perfused, warm, and had normal blood pressure. Control Doppler ultrasonography of the limb showed the AFV was patent and that arterial flow distal of the anastomosis had changed, with attenuation of the velocity curves to biphasic morphology in the popliteal and infrapatellar arteries. The patient remained asymptomatic with normal blood pressure and was discharged from hospital on the second postoperative day.

**Figure 2:**
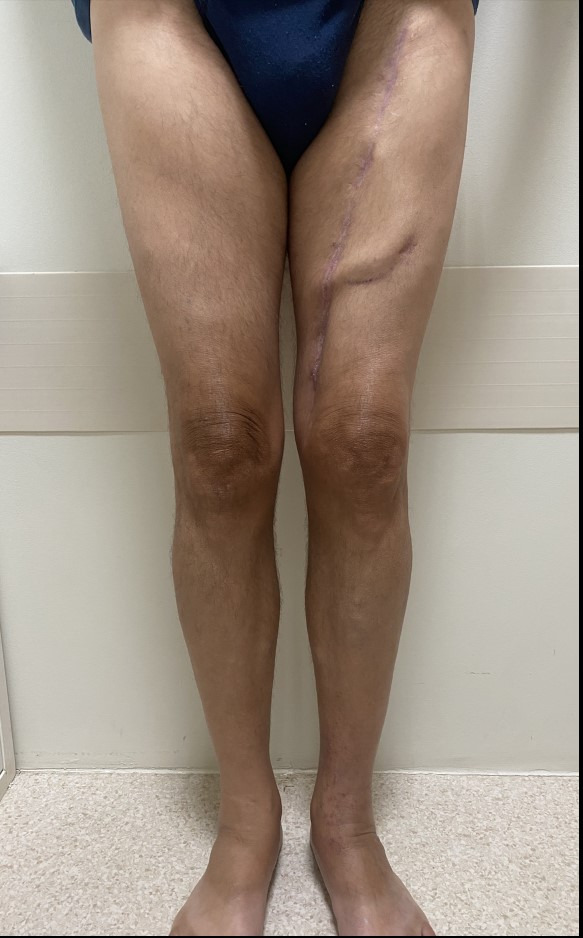
Late postoperative period (3 months). Observe that the lower limbs are free from edema and are symmetrical. The femoral loop is very pronounced under the skin.

**Figure 3:**
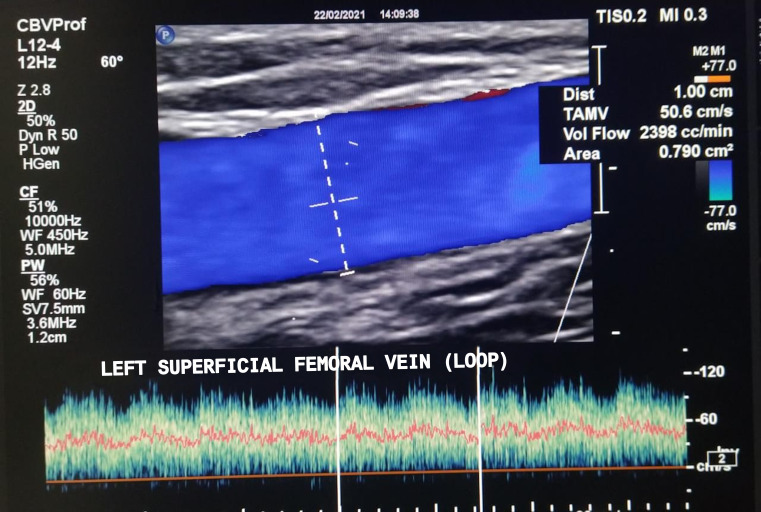
Control Doppler at 3 months. Image showing the 10mm diameter of the superficial femoral vein and flow volume close to 2.4 L/min. Absence of stenosis in the mid venous segment.

Six weeks after the surgery, the AVF began to be used for hemodialysis at the referring clinic (Figure 2). At late postoperative follow-up (3 months), the patient was asymptomatic from an arterial perspective, with no ischemic symptoms and/or claudication of the limb, was free from edema, and was following a regular hemodialysis program. Control Doppler ultrasonography showed the superficial femoral loop AVF was patent, free from stenosis, with a diameter of 10 mm, and blood flow of 2.4 liters/minute (Figure 3). The proximal femoral-iliac venous axis was normal and the popliteal and infrapatellar arteries still had biphasic spectral curves.

## DISCUSSION

A recent analysis demonstrated that there has been a significant increase in the number of chronic kidney disease patients on hemodialysis programs in Brazil over the last decade.^1^ These data, compounded by the unrestricted use of short and long-stay catheters constitute a challenge for nephrologists and vascular surgeons, because of the ever-growing number of patients whose upper-limb access options have been exhausted and the rising rates of central vein stenosis.^3^

The current recommendation is that an upper-limb fistula is the first-choice option for a permanent hemodialysis access.^4^ However, when faced with the challenge of no possible options for upper limb accesses, exceptional techniques can be employed, such as transposition of the brachial vein or creation of an autogenous or prosthetic lower limb AVF, among others.^5^^-^11 Use of a long-dwell catheter in the femoral vein is only indicated in patients with multiple comorbidities and short life expectancy.^4^

Patency rates for SFVt are superior to those achieved with prosthetic loops,^10^ particularly because infections are much more common in lower-limb PTFE loops. In a systematic review comparing PTFE loops in the upper thigh against SFVt, primary patency rates were 48% and 83%, respectively. Similarly, secondary patency rates were 69% and 93%. The rates of infections resulting in loss of access were 18.4% and 1.61%, respectively. However, ischemic complications were more common with the SFVt (20.97% vs. 7.18%).^6^

Gradman et al.^2^ published the first description in the literature of experience with SFVt in 2001. In their retrospective analysis, 18 patients underwent SFVt and there were elevated rates of distal limb ischemia requiring surgical revisits because of ischemic syndrome in eight cases. Despite the excellent primary and secondary patency rates, of 73% and 86% respectively, the authors warned of arterial steal by the AVF as a potential complication associated with the surgical procedure. In practice, steal may not be suspected post operatively and may only appear when the AVF is used.^12^

In the second series published, reported by the same authors in 2005, the incidence of ischemic complications was reduced to zero.^13^ This substantial improvement in relation to the first series was attributed to better patient selection, excluding those with significant peripheral arterial occlusive disease (PAOD). Secondary patency was 94% at 2 years.

The researchers raised important technical questions based on the experience acquired in these two series. Some of their technical recommendations include: extensive dissection of the superficial femoral vein from the popliteal vein in Hunter’s canal as far as its outflow into the common femoral vein to create a long subcutaneous loop; preservation of the deep femoral vein and the great saphenous vein, which will become the limb’s primary drainage route after surgery; and the subcutaneous loop and end-to-side anastomosis in the mid third of the superficial femoral artery. Use of selective banding of the vein at the anastomosis was another technique incorporated in the second group of patients, although little detail was provided.

One of the largest series published in the literature, with excellent medium and long-term follow-up, was described by Bourquelot et al.^5^ in 2012, who published the results of 72 SFVt with primary patency of 91% at 1 year and 45% after 9 years of follow-up. In this series, there were five cases of distal ischemia that required ligature of the AFV (four cases) or limb amputation (one case). All of the patients with ischemic complications were diabetic, suggesting that better patient screening and selection could possibly improve this outcome. In 33 operated patients, flow through the AVF was assessed with Doppler ultrasonography, finding higher volumes than those usually found in AVFs in upper limbs. However, no association was detected between flow through the AVF and ischemic syndrome.

In the case reported here, the patient exhibited loss of distal pulses and attenuated Doppler velocity curves, but remained asymptomatic and with limb blood pressure preserved, and did not require repeat intervention. The risk of arterial ischemia of the limb is the major concern. The importance of careful patient selection is clear, ruling out PAOD, and checking for strong distal pulses and a normal ankle-brachial index, in addition to absence of diabetes. This routine should improve the surgical results, although it limits applications for the method, considering the high prevalence of diabetes mellitus and PAOD among these patients.

In addition to the risk of ischemia, the duration of the procedure, its size, and the details of the dissection involving construction of a loop with the SFVt are other disadvantages of the technique, particularly if the duration of surgery is compared with prosthetic loop construction. These obstacles make SFVt a method for exceptional cases and its indications need to be better studied. Nevertheless, the possibility of ensuring a durable, high-flow, permanent access with a low risk of infection makes SFVt an excellent option in patients for whom few vascular access options remain.

We conducted postoperative follow-up as recommended by the European Society for Vascular Surgery^,4^ with periodic physical examination and control Doppler ultrasonography. Surveillance of the AVF and early intervention improve survival of the access and, consequently, survival of the chronic renal patient.^14^^,^^15^

When upper limb access is not possible, exceptional techniques such as construction of a loop by transposition of the femoral vein can be employed. Although little used, this technique and its results have been well described in the specialist literature and should not be ignored when striving to provide vascular access for these patients.
