# Supplementary material for: Superficial femoral vein transposition as a solution for hemodialysis vascular access
Source: J Vasc Bras. 2022 Oct 3;21:e20210135. doi: 10.1590/1677-5449.202101351 (PMC9536315; doi:10.1590/1677-5449.202101351)
Supplement: Translation

# Translation: Transposição de veia femoral superficial como solução de acesso vascular para hemodiálise

**DOI:** 10.1590/1677-5449.202101352

**Authors:** Matheus Mannarino Carmo Silva Ribeiro, Eduardo Rodrigues, Alex Bezerra, Eric Paiva Vilela, Felipe Borges Fagundes, Cristiane Ferreira de Araújo Gomes, Cristina Ribeiro Riguetti Pinto, Carlos Eduardo Virgini-Magalhães

**Affiliations:** 1 Universidade do Estado do Rio de Janeiro – UERJ, Hospital Universitário Pedro Ernesto – HUPE, Rio de Janeiro, RJ, Brasil.

**Keywords:** fístula arteriovenosa, insuficiência renal crônica, diálise renal

## Abstract

O crescimento significativo do número de indivíduos dependentes de hemodiálise para terapia renal substitutiva e o uso irrestrito de cateteres de curta e longa permanência têm desafiado os cirurgiões vasculares em busca de soluções para a exaustão de acessos nos membros superiores e taxas crescentes de estenose venosa central nesses pacientes. Na impossibilidade do acesso em membros superiores, técnicas excepcionais podem ser utilizadas, e os membros inferiores são uma alternativa factível como sítio de acesso vascular para hemodiálise. Este artigo relata um caso de transposição de veia femoral superficial para confecção de uma fístula arteriovenosa em alça em um paciente sem possibilidade de acessos nos membros superiores, além de fazer uma revisão da literatura sobre essa técnica ainda pouco utilizada.

## INTRODUÇÃO

Na última década, houve um crescimento significativo do número de indivíduos dependentes de hemodiálise para terapia renal substitutiva (TRS). No Brasil, esse número se aproxima rapidamente dos 150.000 pacientes renais crônicos^1^.

Não há dúvida de que as fístulas arteriovenosas (FAVs) autólogas do membro superior são o acesso de escolha para realização da hemodiálise. Entretanto, quando o acesso em membros superiores não é factível, a confecção do acesso vascular no membro inferior torna-se uma alternativa viável e salvadora^2^.

Este trabalho foi aprovado pelo Comitê de Ética em Pesquisa da Instituição, através do parecer 4.783.675 (CAAE: 47569921.3.0000.5259), e tem o objetivo de descrever a confecção cirúrgica de um acesso vascular para hemodiálise através da transposição de veia femoral superficial (tVFS) e, através da revisão de literatura, discutir vantagens e desvantagens da técnica, ainda pouco utilizada em nosso meio.

### Parte 1 – situação clínica

Um paciente do sexo masculino, de 54 anos e em programa de TRS há 21 anos foi encaminhado para confecção de acesso vascular para hemodiálise. Apresentava história de múltiplos acessos nos membros superiores. Ele foi submetido à ligadura de alça de basílica em julho de 2020 devido a estenose venosa central e úlcera com sangramento intermitente na topografia do sítio de punção, sem possibilidade de tratamento endovascular. Negava acessos vasculares provisórios prévios em veias femorais e encontrava-se dialisando via cateter de longa permanência implantado em veia subclávia direita há 2 meses.

Ao exame físico vascular, apresentava múltiplas cicatrizes de FAVs prévias em membros superiores, sem possibilidade de confecção de novo acesso por ausência de segmento venoso viável para a cirurgia e estenose venosa central à esquerda.

Diante da impossibilidade dos membros superiores, o dilema que se apresentava seria a confecção do acesso vascular no membro inferior, utilizando-se material autólogo ou protético.

### Parte 2 – O que foi feito

Optamos pela transposição da veia femoral, considerando os melhores resultados de perviedade e baixo risco de infecção, em comparação com a alça de veia safena ou a utilização do enxerto de politetrafluoretileno (PTFE) para esse objetivo, e a anatomia arterial e venosa adequada dos membros inferiores do paciente, com pulsos arteriais amplos, índices tornozelo-braquial normais e sistema venoso superficial e profundo sem alterações ao eco-Doppler.

O paciente foi submetido à transposição de veia femoral superficial em membro inferior esquerdo em novembro de 2020, sob raquianestesia conforme técnica descrita por Gradman et al.^2^. A veia safena magna foi identificada com o eco-Doppler imediatamente antes da cirurgia, para prevenir sua lesão durante a realização do acesso. O procedimento ocorreu sem intercorrências intraoperatórias, com cerca de 2,5 horas de duração (Figura 1).

**Figura 1:** Procedimento cirúrgico. Detalhe da anastomose entre artéria e veia femoral superficial.

No primeiro dia de pós-operatório, evidenciou-se redução significativa da palpação dos pulsos poplíteo e distais ao exame físico, apesar de o membro estar bem perfundido, aquecido e compensado. O eco-Doppler de controle do membro demonstrou perviedade da FAV e alteração do fluxo arterial distal à anastomose com atenuação das curvas de velocidade para morfologia bifásica em artérias poplítea e infrapatelares. O paciente permaneceu assintomático e compensado, recebendo alta hospitalar no segundo dia de pós-operatório.

Após 6 semanas de cirurgia, iniciou-se a utilização da FAV para hemodiálise na clínica de origem (Figura 2). No seguimento pós-operatório tardio (3 meses), o paciente encontrava-se assintomático do ponto de vista arterial, sem sintomas isquêmicos e/ou claudicação do membro, sem edema e em programa de hemodiálise regular. Um novo eco-Doppler de controle evidenciou FAV em alça de femoral superficial pérvia, sem estenoses, com 10 mm de diâmetro e volume de fluxo de 2,4 litros/minuto (Figura 3). O eixo venoso femoral proximal e ilíaco não apresentou alterações, e as artérias poplítea e infrapatelares permaneciam com curva espectral bifásica.

**Figura 2:** Pós-operatório tardio (3 meses). Observam-se os membros inferiores sem edemas e simétricos. Alça de femoral bem pronunciada sob a pele.

**Figura 3:** Doppler de controle de 3 meses. Imagem mostrando diâmetro da veia femoral superficial de 10 mm e volume de fluxo próximo de 2,4 L/min. Ausência de estenoses no segmento médio venoso.

## DISCUSSÃO

Um levantamento recente constatou aumento significativo de pacientes com doença renal crônica em programa de hemodiálise na última década no Brasil^1^. Esses dados, associados ao uso irrestrito de cateteres de curta e longa permanência, impõem um desafio para nefrologistas e cirurgiões vasculares, porque cada vez mais pacientes apresentam-se com exaustão de acessos nos membros superiores e taxas crescentes de estenose venosa central^3^.

A recomendação atual é que a fístula em membro superior seja a primeira escolha para o acesso permanente para hemodiálise^4^. No entanto, diante do desafio da impossibilidade do acesso em membros superiores, técnicas excepcionais podem ser utilizadas, como a transposição do veia braquial, a confecção de FAV no membro inferior autógena ou com prótese, entre outras^5^^-^11. A utilização de cateter de longa permanência em veia femoral só é indicada em pacientes com múltiplas comorbidades e baixa expectativa de vida^4^.

As taxas de perviedade da tVFS são muito superiores quando comparadas às de alça com prótese^10^, em especial porque a infecção em alças de PTFE nos membros inferiores são muito mais comuns. Em uma revisão sistemática comparando alça de PTFE em coxa superior vs. tVFS, a perviedade primária foi de 48% e 83%, respectivamente. Da mesma forma, a perviedade secundária foi 69% e 93%. Já a infecção que resultou em perda do acesso obteve taxas de 18,4% e 1,61%, respectivamente. Por outro lado, as complicações isquêmicas foram mais comuns na tVFS (20,97% vs. 7,18%)^6^.

A primeira descrição na literatura da experiência com tVFS foi realizada por Gradman et al.^2^ e publicada em 2001. Nessa análise retrospectiva, 18 pacientes foram submetidos à tVFS, sendo identificadas elevadas taxas de isquemia distal de membro e necessidade de reabordagem cirúrgica devido a síndrome isquêmica em oito casos. Apesar de excelentes taxas de perviedade primária e secundária, 73% e 86%, respectivamente, os autores alertaram para o roubo arterial pela FAV como um potencial complicador associado ao procedimento cirúrgico. Na prática, o roubo pode não ser suspeitado no pré-operatório e surgir somente com a utilização da FAV^12^.

Na segunda série relatada pelos mesmos autores em 2005, a incidência de complicações isquêmicas declinou para zero^13^. Essa melhora substancial em relação à primeira série foi atribuída à melhor seleção de pacientes, excluindo indivíduos com doença arterial obstrutiva periférica (DAOP) significativa. A perviedade secundária encontrada foi de 94% em 2 anos.

Com a experiência adquirida nessas duas casuísticas, os pesquisadores ressaltaram questões técnicas importantes. Alguns conselhos técnicos descritos são estes: a dissecção ampla da veia femoral superficial desde a veia poplítea no canal de Hunter até a sua desembocadura na veia femoral comum para realizar uma alça longa no subcutâneo; a preservação da veia femoral profunda e da veia safena magna, que se transformam na principal via de drenagem venosa no membro após a cirurgia; e a alça no subcutâneo e anastomose terminolateral no terço médio da artéria femoral superficial. A utilização de uma bandagem seletiva na veia justa anastomótica foi outra incorporação técnica descrita no segundo grupo de pacientes, embora sem muitos detalhes.

Uma das maiores séries publicadas na literatura, com um excelente acompanhamento de médio e longo prazo, foi descrita por Bourquelot et al.^5^ em 2012, quando publicaram resultados de 72 tVFS realizadas com perviedade primária de 91% em 1 ano e de 45% em 9 anos de acompanhamento. Nessa série, houve cinco casos de isquemia distal que necessitaram ligadura da FAV (quatro casos) e amputação do membro (um caso). Todos os pacientes com complicações isquêmicas eram diabéticos, sugerindo que possivelmente uma melhor triagem e seleção dos pacientes possa melhorar esse desfecho. Em 33 pacientes operados, o fluxo na FAV foi avaliado pelo eco-Doppler, que encontrou volumes maiores aos encontrados usualmente nas FAVs de membros superiores. No entanto, não foi constatada associação do fluxo pela FAV com síndrome isquêmica.

No caso relatado aqui, o paciente apresentou perda de pulsos distais e amortecimento das curvas de velocidade ao Doppler, porém manteve-se assintomático e com o membro compensado, sem necessidade de nova intervenção. O risco de isquemia arterial do membro é a grande preocupação. Fica clara a importância de uma seleção cuidadosa do paciente com exclusão de DAOP, presença de pulsos distais amplos e índice tornozelo-braquial normal, além de ausência de diabetes. Essa rotina deve melhorar os resultados cirúrgicos, embora possa limitar a utilização do método, considerando a grande prevalência do diabetes melito e da DAOP entre esses pacientes.

Além do risco de isquemia, a duração do procedimento, sua extensão e os detalhes da dissecção que envolvem a confecção da alça com tVFS são outras desvantagens da técnica, em especial se compararmos os tempos operatórios com a confecção da alça protética. Esses óbices fazem da tVFS um método de exceção, e sua indicação precisa ser bem avaliada. Ainda assim, a possibilidade de garantir um acesso permanente durável, de alto fluxo e com baixo risco de infecção torna a tVFS uma excelente alternativa a pacientes quando restam poucas opções de acesso vascular.

Realizamos o seguimento pós-operatório como recomendado pela Sociedade Europeia de Cirurgia Vascular^4^, com exame físico e eco-Doppler de controle periódicos. A vigilância da FAV e a intervenção precoce aumentam a sobrevida do acesso e, consequentemente, a sobrevivência do próprio paciente renal crônico^14^^,^^15^.

Na impossibilidade do acesso em membros superiores, técnicas excepcionais, como a confecção de uma alça através da transposição da veia femoral, podem ser utilizadas. Embora pouco utilizada, essa técnica e seus resultados já foram bem descritos na literatura especializada e não devem ser ignorados, no esforço de garantir um acesso vascular para esses pacientes.
